# Characterization of terpene biosynthesis in *Melaleuca quinquenervia* and ecological consequences of terpene accumulation during myrtle rust infection

**DOI:** 10.1002/pei3.10056

**Published:** 2021-06-24

**Authors:** Ji‐Fan Hsieh, Sandra T. Krause, David Kainer, Jörg Degenhardt, William J. Foley, Carsten Külheim

**Affiliations:** ^1^ Research School of Biology The Australian National University Canberra ACT Australia; ^2^ Institut für Pharmazie Martin‐Luther Universität, Halle‐Wittenberg Halle Germany; ^3^ Center for BioEnergy Innovation Bioscience Division Oak Ridge National Laboratories Oak Ridge TN USA; ^4^ College of Forest Resources and Environmental Science Michigan Technological University Houghton MI USA

**Keywords:** broad‐leaved paperbark, essential oil, myrtle rust, terpene synthase

## Abstract

Plants use a wide array of secondary metabolites including terpenes as defense against herbivore and pathogen attack, which can be constitutively expressed or induced. Here, we investigated aspects of the chemical and molecular basis of resistance against the exotic rust fungus *Austropuccinia psidii* in *Melaleuca quinquenervia*, with a focus on terpenes. Foliar terpenes of resistant and susceptible plants were quantified, and we assessed whether chemotypic variation contributed to resistance to infection by *A. psidii*. We found that chemotypes did not contribute to the resistance and susceptibility of *M. quinquenervia*. However, in one of the chemotypes (Chemotype 2), susceptible plants showed higher concentrations of several terpenes including α‐pinene, limonene, 1,8‐cineole, and viridiflorol compared with resistant plants. Transcriptome profiling of these plants showed that several *TPS* genes were strongly induced in response to infection by *A. psidii*. Functional characterization of these *TPS* showed them to be mono‐ and sesquiterpene synthases producing compounds including 1,8‐cineole, β‐caryophyllene, viridiflorol and nerolidol. The expression of these *TPS* genes correlated with metabolite data in a susceptible plant. These results suggest the complexity of resistance mechanism regulated by *M*. *quinquenervia* and that modulation of terpenes may be one of the components that contribute to resistance against *A. psidii*.

## 1 INTRODUCTION

Terpenes, a class of secondary metabolites, play a key role in both constitutive and induced defenses in many plants. Terpene diversity arises from a large family of enzymes called terpene synthases (TPS; Degenhardt et al., 2009), which fold prenyl diphosphate substrates, such as farnesyl diphosphate (FPP) and geranyl diphosphate (GPP) into terpenes (Bohlmann et al., 1998; Köllner et al., 2004). GPP is produced through the 2‐C‐methyl‐D‐erythritol 4‐phosphate (MEP) pathway in the chloroplast, whereas FPP is produced through the mevalonate (MVA) pathway in the cytosol. GPP is the precursor of monoterpenes with 10 carbon units, and FPP is used as a precursor to sesquiterpenes with 15 carbon units (Chen et al., 2011).

In contrast to studies of terpenes in plant–insect interactions, their role in fungal infections is less well known. Other than studies that investigated the effects of terpenes to beetle‐vectored fungal defense in several conifer species (Keeling & Bohlmann, 2006; Zeneli et al., 2006), only few studies investigated induced terpenes with potential antifungal properties in plants in response to fungal infections alone. For example, Visser et al. (2015) compared gene expression profiles between resistant and susceptible *Eucalyptus* *grandis* in response to the necrotrophic fungus *Chrysoporthe austroafricana* and identified inductions of transcripts encoding a putative isoprene synthase (*EgrTPS084*) and a β‐caryophyllene synthase (*EgrTPS038*) after infection in resistant plants. Maize (*Zea mays*) responds to infection by *Fusarium graminearum* by producing zealexins and kauralexins (both terpenoids) in response to *Cercospora zeina* (Huffaker et al., 2011; Meyer et al., 2017). Similarly, bell pepper (*Capsicum annuum*) accumulates the terpenoid capsidiol, which affects the growth of fungal‐like oomycete pathogens *Phytophthora capsici* and *Phytophthora* *infestans* (Giannakopoulou et al., 2014).

The recent invasion of the exotic rust fungus *Austropuccinia psidii* (G. Winter) Beenken *comb*. *nov*. (Beenken, 2017; myrtle rust) in Australia has infected hundreds of woody plant species in the Myrtaceae (Carnegie et al., 2016). The rust is a serious biosecurity concern as species of Myrtaceae dominate much of the Australian flora (Ladiges et al., 2003) and include many species‐rich genera, such as *Eucalyptus* and *Melaleuca*, which are widely utilized for their terpene‐dominated essential oils (Boland et al., 1991; Carson et al., 2006) and are globally planted for pulp and hardwood production (Myburg et al., 2014). This rust disease has severely affected several industries that depend on Myrtaceae (Carnegie et al., 2016) and may threaten the biodiversity of Australia, particularly along the east coast wetlands (Pegg et al., 2014). *Melaleuca quinquenervia* is one of the species most at risk. It is the dominant woody plant species of the east coast wetland ecosystem (Barlow, 1988; Cook et al., 2008). Field studies on *M. quinquenervia* have observed foliar and stem dieback, as well as flower death, which suggests that the disease may lead to disruptive long‐term consequences to ecological function of wetlands as the symptoms may impede forest regeneration (Pegg et al., 2014).

**FIGURE 1:**
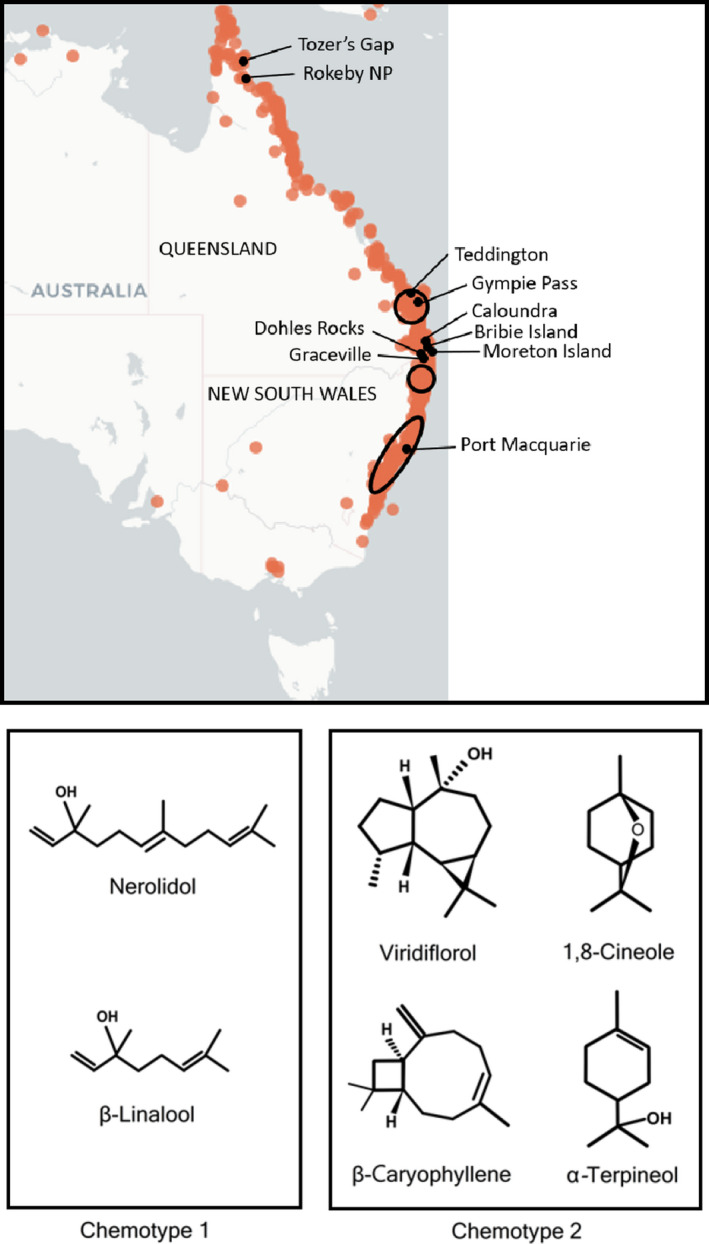
(a) Approximate geographic distribution of Chemotype 1 and 2 *Melaleuca quinquenervia* in Australia. Orange dots represent distribution of *M. quinquenervia*. Chemotype 1 occurs mostly in circled areas (black), whereas Chemotype 2 is distributed throughout the entire range of the species. The distribution of Chemotype 1 was derived from data in Ireland et al. (2002); map was produced using The Atlas of Living Australia. Seed source populations are indicated on map. (b) Major types of terpenes found in Chemotypes 1 and 2 of *M. quinquenervia* (figure design adapted from Padovan et al., 2010)

Screening studies showed *M. quinquenervia* was variably resistant to *A. psidii* (Hsieh et al., 2017; Morin et al., 2012). *M. quinquenervia* displays a high degree of qualitative variability in foliar essential oils (Ireland et al., 2002) described as “chemotypes” (chemical phenotypes). Different chemotypes of *M. quinquenervia* have characteristic terpene profiles, which have been suggested to play important roles in ecology, such as in host plant selection by insects (Padovan et al., 2010; Wheeler & Ordung, 2005). Two chemotypes have been identified (Ireland et al., 2002). Chemotype 1 occurs mainly in the southern range of the species, including Sydney (33°54′S, 151°15′E), Selection Flat (29°09′S 152°59′E), and Maryborough (25°33′S, 152°42′E) with a terpene profile dominated by nerolidol and β‐linalool (Figure 1b). Chemotype 2 is distributed across the geographic range of the species (Figure 1a) and is dominated by viridiflorol and 1,8‐cineole with smaller amounts of β‐caryophyllene and α‐terpineol (Figure 1b).

In a previous study (Hsieh et al., 2017), we observed many mRNA transcripts from defense‐related genes that were upregulated after *A. psidii* infection in the transcriptome of *M. quinquenervia*. Many transcripts showed strong, multifold inductions in some resistant but not all individuals and were hence not significant among biological replicates. These included several transcripts encoding putative TPS enzymes. The identification of resistant and susceptible individuals of *M. quinquenervia* to *A. psidii*, together with information on chemotypic variation of terpenes in *M. quinquenervia*, provides an opportunity to study the role of terpenes in woody plant species in defense against rust disease. The aim of this study was therefore to incorporate both chemical and molecular approaches to investigate the effect of terpene variation and terpene regulation by *TPS* genes that may have contributed to the resistance of *M. quinquenervia* plants against infection by *A. psidii*. We first assessed whether chemotypic variation contributed to the resistance and susceptibility of *M. quinquenervia* against *A. psidii*. Subsequently, we used transcriptome profiling to mine putative *TPS* transcripts and selected candidate transcripts that were induced in response to rust. We then functionally characterized several of these *TPS* genes which had been induced in response to fungal infection.

## 2 MATERIALS AND METHODS

### 2.1 Plant material and rust inoculation

Sixty‐two *M. quinquenervia* plants were grown from seed purchased from the Australian Tree Seed Centre (CSIRO), sourced from ten populations across eastern Australia ranging from Tozers Gap (13°44′S, 143°11′E) to Port Macquarie (31°30′S, 152°40′E; Figure 1a). Plants were pruned and allowed to produce fresh growth required for *A. psidii* inoculations. Plants were inoculated at the Plant Breeding Institute, University of Sydney, NSW, together with young susceptible *Syzygium jambos* plants as positive control. A rust spore suspension of 2 mg urediniospores in 1 ml of light mineral oil was sprayed over young leaves. Plants were then incubated for 24 h at 20℃ and high (>95%) relative humidity. Plants were then moved to climate controlled naturally lit rooms and kept at 22℃. Pustules were scored 14 days’ postinoculation on a six‐category scale from highly resistant (HR) to highly susceptible (HS). Inoculations and scoring were done twice to ensure consistent phenotypes. Of the 62 plants, 15 were highly resistant (HR), six were resistant (R), three were moderately resistant (MR), five were moderately susceptible (MS), 20 were susceptible (S), and 13 were highly susceptible (HS). Three of the HR and four of the HS plants had published RNAseq data (Hsieh et al., 2017).

### 2.2 Identification of differentially expressed *TPS* and terpene biosynthesis‐related transcripts in *M. quinquenervia*

From the transcriptome profiling study of four highly resistant (HR) and four highly susceptible (HS) *M. quinquenervia* in response to *A. psidii* (Hsieh et al., 2017), we observed that the expression of defense‐related genes in response to *A. psidii* were highly dissimilar among biologically independent samples of HR. Several of these variable yet nonsignificant transcripts (compared with susceptible) encoded *TPS*. As such inductions may be biologically important and remembering that our samples are genetically diverse, we selected those transcripts encoding *TPS* (hereafter referred to as “*TPS* transcripts”) and calculated fold changes (FC) of transcripts per million (TPM) for each genotype individually, which showed high levels of induction in one of the genotypes. Details of RNA extraction and sequencing of these samples are described in Hsieh et al. (2017).

### 2.3 Phylogenetic analysis of *M. quinquenervia TPS* compared with other Myrtaceae

Translated amino acid sequences of all *TPS* transcripts observed from the *M. quinquenervia* transcriptome (Hsieh et al., 2017) were retrieved to align against known *E*. *grandis* TPS sequences with subfamily information (Külheim et al., 2015). Two characterized *M. quinquenervia* viridiflorol synthases (Padovan et al., 2010), one *M. alternifolia* isoprene synthase (Genbank accession AY279379; Sharkey et al., 2013), one *M. alternifolia* terpinolene synthase, one *M. alternifolia* sabinene hydrate synthase, and two *M. alternifolia* 1,8‐cineole synthases (Padovan et al., 2017) were also included in the phylogenetic analysis to assess the homology of *TPS* transcripts to known terpene synthases from *Melaleuca*.

Before aligning *M. quinquenervia TPS* sequences to those of *E. grandis* and *M. alternifolia*, they were first manually aligned with the characterized *M. quinquenervia* viridiflorol synthase gene sequences using BioEdit (Version 7.2.6; Hall, 1999) to check that sequences were not identical or potential allelic variants of each other. Sequences were aligned using conserved diagnostic motifs as anchors. These motifs include: RRX_8_W (the cap of the catalytic pocket), RLLR (situated in the latter half of the first exon), YEACH/LEASH, RWW/RWG/RWV (intron–exon boundary), DDXXD (involved in Mg^2+^‐assisted substrate binding), and (N,D)DX_2_(S,T)X_3_E. Sequences that were identical or varied only by length differences were discarded (keeping the longest).

Subsequently, we manually aligned the *M. quinquenervia TPS* sequences to 113 previously aligned *E. grandis* TPS sequences described by Külheim et al. (2015) and the five functionally characterized *M. alternifolia* sequences. All sequences were then truncated as described (Külheim et al., 2015). PhyML (Version 3.0; Guindon et al., 2010) was used to determine phylogenetic relationships between the sequences based on maximum likelihood (ML). The Jones–Taylor–Thornton (JTT) substitution model was used as it had the lowest AICc value (Akaike's information criterion value, corrected for sample size). Node support was calculated using 100 bootstrap replicates in PhyML.

Some sequences derived from the transcriptome were too short for use in the phylogeny and were removed if they did not cover any of the conserved motifs. Further, pairwise distances between all sequences were first estimated by MEGA7 (Kumar et al., 2016), and sequences which were too fragmented were removed. An initial tree was then estimated by the neighbor‐joining (NJ) method in MEGA7 before building the phylogeny by ML in PhyML. The final tree from PhyML was visualized using FigTree (Version 1.4.3; Rambaut, 2010). In the phylogenetic tree, three transcripts (Mq50098c0s1, Mq50098c0s12, and Mq50098c0s20) were identical but were retained in the analysis because these sequences (in translated amino acids) were different before truncation. Sequence identities were determined by BioEdit (Version 7.2.6; Hall, 1999) with BLOSUM62 similarity matrix.

### 2.4 *TPS* transcripts for functional characterization

Full‐length or near‐full‐length *TPS* transcripts which showed induction (FC >2) in one or more *M. quinquenervia* individuals after *A. psidii* infection were considered for functional characterization. A final list of eight candidate sequences was chosen for characterization based on the phylogenetic analysis, which categorized the sequences into subfamilies (TPS‐a, TPS‐b, TPS‐b2, and TPS‐g).

RNA from one highly susceptible (HS1) and one highly resistant (HR1) *M. quinquenervia* sample (Hsieh et al., 2017) were used for functional characterization of *TPS*. Single‐stranded cDNA was synthesized using the Fermentas First Strand cDNA Kit (MBI Fermentas) following the manufacturer's instructions. The candidate *TPS* sequences were first isolated and amplified from cDNA using designed primers (Table S1). The PCR fragments were then cloned into the pCR4‐TOPO vector (Thermo Fisher Scientific), which were then introduced into TOP10 *Escherichia coli* cells (Invitrogen) for sequencing to ensure that the correct sequences were isolated and no PCR‐related errors were introduced.

Subsequently, fragments were subcloned into the pASK‐IBA37+ expression vector (IBA GmbH) by reamplifying the fragments with primers designed to have overhangs specific for the pASK‐IBA37+ vector (Table S1). Additionally, some of the fragments had forward primers designed at the RRX_8_W motif so that signal peptide regions were not amplified. Heterologous expression and characterization were done following the procedures described by Külheim et al. (2015). Briefly, TOP10 *E. coli* cells with the pASK‐IBA+ expression vector were cultured at 37℃ to a density (OD_600_) of 0.5–0.6. The expression vector was then induced with anhydrotetracycline. The cell culture was harvested after 20‐h incubation (18℃) by centrifugation, which was then disrupted by four cycles of 30‐s sonication at 50% amplitude (Branson Sonifier 250) while in chilled extraction buffer (50 mM Tris–HCl pH 7.5, 5 mM Na‐ascorbate pH 7.0, 5 mM MgCl_2_, 5 mM dithiothreitol (DTT), 0.5 mM phenylmethylsulfonyl fluoride, and 10% glycerol). After centrifugation at 14,000 *g* to separate the cell debris from the crude protein extract, the supernatant containing the extract was transferred into the assay buffer (10 mM Tris–HCl pH 7.5, 10% glycerol, and 1 mM DTT) using an Econo‐Pac 10DG column (Bio‐Rad Laboratories).

Terpene synthase activity assays were done by first adding 30 μl of crude enzyme extract, 13.2 ng/μl GPP or (E,E)‐FPP, 10 mM MgCl_2_, and 58 μl of assay buffer into a glass gas chromatography (GC) assay tube (Macherey‐Nagel). Products from the enzymes were collected using a solid‐phase microextraction fiber (SPME) of 100 μm polydimethylsiloxane (Supelco)—the SPME was exposed in the headspace of the assay tube during incubation of the mixture (45 min at 35℃). The SPME was then placed into the injector of the GC (GC‐2010, Shimadzu). Terpenes were separated by the GC using an EC‐5 (Grace) column (5% phenyl methylpolysiloxane, with a length of 30 m, an internal diameter of 0.25 mm, and a film thickness of 0.25 μm). Splitless injection was used at an injector temperature of 220℃; hydrogen was used as the carrier gas at 1 ml min^−1^. The temperature program for monoterpenes was set as follows: Hold at 50℃ for 3 min, then ramp up to 150℃ at 7℃ min^−1^, followed by another ramp to 300℃ at 100℃ min^−1^, and hold for 2 min. The temperature program for sesquiterpenes was as follows: Hold at 80℃ for 3 min, then ramp up to 200℃ at 7℃ min^−1^, then up to 300℃ at 100℃ min^−1^, and then hold for 2 min. Terpenes were identified by a mass spectrometry (MS; GCMS‐QP 2010 Plus, Shimadzu) attached to the GC and “GCMS Postrun Analysis” (Shimadzu software) with the MS library “Wiley8” (Hewlett‐Packard) to determine the identity of the terpenes. The two viridiflorol synthases were further validated by authentic standards (Sigma‐Aldrich) as previous GC profiles gave similarly high NIST scores for both aromadendrene and viridiflorol.

### 2.5 GC‐MS profiling of foliar terpenes

Sixty‐two *M. quinquenervia* plants were used for terpene profiling before, and 5 days’ postinoculation (dpi), with *A. psidii* spores. Terpenes from the seven plants used for RNAseq (Hsieh et al., 2017) were extracted with ethanol from ground leaf powder stored at −80℃, whereas terpenes from the remaining 110 samples from 55 plants were extracted by immersing fresh leaf in ethanol as described below.

Approximately 0.4 g of leaf was placed into pre‐weighed 7‐ml glass vials which contained 5 ml of ethanol with tetradecane (0.25 g L^−1^) added as internal standard (IS). Samples were left in the ethanol/tetradecane solvent for 7 days before analysis of terpene profiles by gas chromatography and mass spectrometry (GC‐MS) on an Agilent 6890 GC (Agilent Technologies) using an SGE BPX‐35 (35% phenyl polysilphenylene‐siloxane) column (Trajan Scientific Australia Pty Ltd.). The column was 60 m long, with an internal diameter of 0.25 mm and a film thickness of 0.25 μm. Helium was used as the carrier gas. To prepare the samples for GC‐MS, the solvent extracts were decanted into autosampler vials; for sample solutions with leaf powder sediments, syringe filters (pore size 0.45 μm) were used to remove leaf fragments. One μl of the sample was injected with an autosampler (Agilent/HP 7683) at 250℃ at a 1:25 split ratio. The temperature program was set as follows: 100℃ for 5 min, ramping to 200℃ at 20℃ min^−1^, then up to 250℃ at 5℃ min^−1^, and holding at 250℃ for 4 min (total elution time: 25 min). Mass spectra were acquired using a mass selective detector (Agilent/HP 5973) at an ion source temperature of 250℃, quadrupole temperature of 150℃, and transfer line temperature of 280℃ with ion source filament voltage of 70 eV.

Peaks in the chromatograph were identified by comparing the mass spectra to those in the National Institute of Standards and Technology (NIST) MS library installed in the automated mass spectral deconvolution and identification system (AMDIS) software (Stein et al., 2002); compounds were also identified by comparison to peaks in published studies which had used the same chromatographic conditions (Padovan et al., 2010; Southwell & Stiff, 1990). To automate peak identification and quantification for all samples, we used *easyGC* (GitHub: https://github.com/dkainer/easyGC), which is an analysis pipeline using the PyMS python library (Kainer et al., 2017; O'Callaghan et al., 2012) that quantifies peaks from mass spectra in comparison with our IS (dodecane). Parameters for *easyGC* to call peaks were as follows: –W (widen peak width to capture more ions) was 13, –N (minimum ion count to allow NIST calls) was 3, and –M (“noisemult,” the sensitivity threshold for a peak to be called) was 1.8. The parameters for cross‐sample peak alignment were default, and the range of retention time for peak calling was set from 6.5 to 23 min, as this range included all peaks based on observations in manual identifications.

### 2.6 Terpene variation in response to *A. psidii* infection

Principal component analyses (PCA) of the terpene profiles from the 62 plants, both before and after *A. psidii* infection, were performed using “ggfortify” in R (Version 3.4.0) in order to identify chemotype clusters. Welch's *t*‐test was used to investigate whether resistant plants had a significantly different terpene profile than susceptible plants. For the statistical test, the original six categories were reduced to two, resistant (RES) and susceptible (SUS). To assess proportional up‐ and downregulation of terpenes in resistant (RES) and susceptible (SUS) plants of Chemotypes 1 and 2 in response to *A. psidii* infection, the change of terpene concentrations in each plant sample at 5 dpi was calculated. In addition, a two‐way ANOVA was used to test for changes of terpenes in *M. quinquenervia* plants before and after *A. psidii* infection, as well as between resistant and susceptible plants.

## 3 RESULTS

### 3.1 Identification of induced *TPS* genes of *M. quinquenervia* in response to *A. psidii* infection and functional characterization

To identify terpene synthase genes (*TPS*) that may be inducible by *A. psidii* in resistant and susceptible *M. quinquenervia*, we investigated the expression profiles of *TPS* from the RNASeq transcriptome data of four highly susceptible (HS) and four highly resistant (HR) *M. quinquenervia*. Among the 57 putative *TPS* transcripts observed in the transcriptome of *M. quinquenervia*, eight full‐length or near‐full‐length *TPS* transcripts showed a strong induction in HS or HR plants at 5 dpi (Table S2) and were thus selected for functional characterization to identify the terpenes they produced.

**FIGURE 2:**
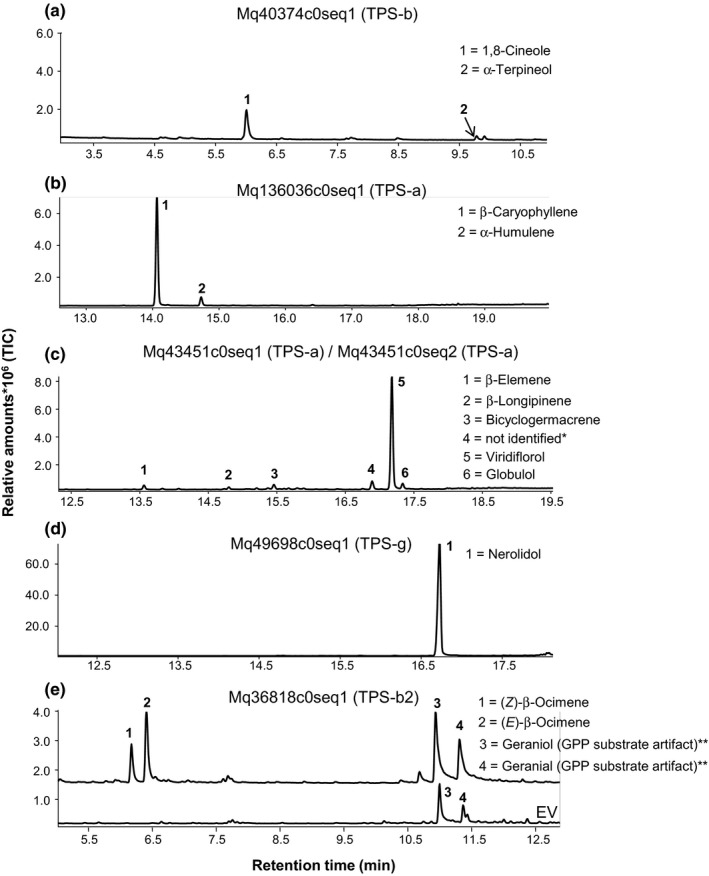
Gas chromatographic (GC) profile of terpene products identified from in vitro functional characterization of six candidate *Melaleuca quinquenervia TPS* genes which were induced in response to *Austropuccinia psidii* infection (5 dpi). Mq43451c0seq1 and Mq43451c0seq2 had the same profiles, and only the first is shown. The GC trace of terpene products from Mq36818c0seq1 is shown in comparison with the empty vector controls (EV). *Best hits: α‐muurolene, germacrene D, germacrene D‐4‐ol; **bacterial enzyme products observed in *Escherichia coli* with an empty expression vector

Of the eight *TPS* candidates, we successfully characterized six terpene synthases. Product profiles from all six terpene synthases showed them to be dominated by one type of terpene (Figure 2). The terpene synthases were thus characterized as a 1,8‐cineole synthase, a β‐caryophyllene synthase, and two viridiflorol synthases. The first acyclic terpene synthase from the Myrtaceae family, a nerolidol synthase, was also identified. A terpene synthase (Mq36818c0seq1) producing β‐ocimene (which is classified as TPS clade b2) was characterized without signal peptide and with GPP as substrate, although only traces of products were present in the GC profile (Figure 2). The remaining two candidate *TPS* genes, Mq46296c0seq1 and Mq53793c0seq7, were also successfully isolated. Mq53793c0seq7 was inactive after attempts to subclone with a signal peptide‐truncated version of the sequence and a change of substrate (to FPP), whereas Mq46296c0seq1 could not be verified by sequencing nor cloned into a vector.

### 3.2 Amino acid alignment of functionally characterized *TPS* genes

Aligned amino acid sequences of functionally characterized *TPS* genes showed that sequences had conserved motifs indicative of terpene synthase functions (Figure S1). Notably, the nerolidol synthase was shorter than other sequences, in that it lacked the common RRX_8_W motif. One of the two viridiflorol synthases characterized here, Mq43451c0s1_Vir, had only one amino acid difference (positioned at 328) to a previously characterized viridiflorol synthase, MqTPS1_Vir (Figure S1).

In addition, we compared translated amino acid sequence identities between transcriptome‐derived sequences and those derived from cloning to check for potential differences. Sequences from the six functionally characterized *TPS* genes were evaluated, and all *TPS* genes other than Mq43451c0seq1_Vir had amino acid sequence identities of 99.1%–100%. For Mq43451c0s1_Vir, the sequence that was cloned had an amino acid identity of 94.9% to the original transcriptome‐derived sequence. Due to high amino acid similarities between members of the TPS family, which often encode *TPS* genes that are identical in function, the primers that intended to amplify the transcriptome‐derived sequence may have amplified the allelic variant of a previously characterized viridiflorol synthase. The cloned sequence was instead more similar (99.7%) to another characterized viridiflorol synthase (Padovan et al., 2010).

### 3.3 Phylogenetic analysis of *TPS* genes of *M. quinquenervia* compared with *E. grandis* and *M. alternifolia*

**FIGURE 3:**
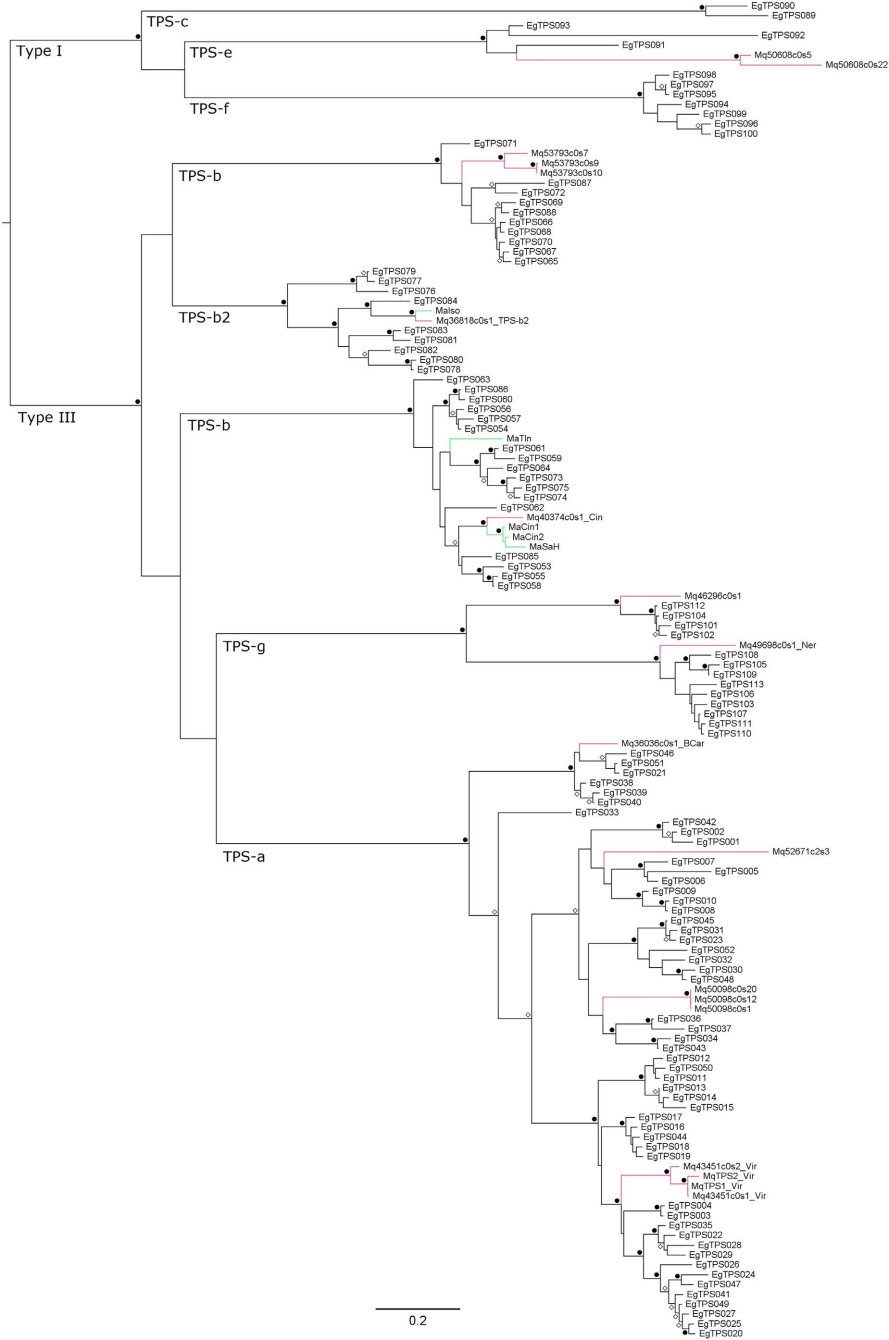
Phylogenetic analysis of *TPS* transcripts of *Melaleuca quinquenervia* (pink branches) with *TPS* genes of *Eucalyptus grandis* and characterized *TPS* genes of *M. alternifolia* (green branches). The tree was constructed by maximum likelihood in PhyML with 100 bootstraps. Nodes with bootstrap values ≥95% and ≥80% are noted by black circles (⚫) and white diamonds (◇), respectively. The scale bar represents 0.2 substitutions per site

After removing short or incomplete transcript fragments observed in the *M. quinquenervia* transcriptome, 16 full‐length or near‐full‐length putative *M. quinquenervia TPS* transcripts were examined for their phylogenetic relationships with *TPS* genes from *E. grandis* and known characterized *TPS* genes of *M. alternifolia* (Figure 3). The phylogeny used the sequences from the six functionally characterized genes rather than their transcriptome‐derived sequences. The phylogenetic analysis confirmed that one of the characterized viridiflorol synthases, Mq43451c0s2_Vir, was different from previous characterized *M. quinquenervia* viridiflorol synthases (MqTPS1_Vir and MqTPS2_Vir) by Padovan et al. (2010). On the other hand, Mq43451c0s1_Vir appeared to be an allelic variant of MqTPS1_Vir (Figure 3).

We also discovered *TPS* transcripts that belonged to the TPS‐e, TPS‐b, TPS‐b2, and TPS‐g subfamilies, respectively (Figure 3). In one of the TPS‐b clades, the *M. quinquenervia* transcript Mq40374c0s1_Cin characterized as a 1,8‐cineole synthase was sister to the *M. alternifolia* clade that included functionally characterized 1,8‐cineole synthases and sabinene hydrate synthase. The *M. quinquenervia* transcript Mq49698c0seq1_Ner resided in clade TPS‐g, which corresponded to characterization results of a nerolidol synthase (TPS‐g).

Notably, the *TPS* transcript (Mq36818c0seq1) of *M. quinquenervia* that was characterized with traces of β‐ocimene in the GC product profile formed a monophyletic group with isoprene synthases characterized in *M. alternifolia* (MaISO; Genbank #AY279379; Sharkey et al., 2013) and predicted in *E. grandis* (TPS084; Külheim et al., 2015). For this reason, we aligned the three sequences to check whether Mq36818c0seq1 contained amino acid residues canonical to isoprene synthases. The Mq36818c0seq1 contained four specific amino acids, FSFN as in other functionally characterized isoprene synthases (Figure S2). Neither substrate nor GC methods used in this study allowed us to test whether this gene is a functional isoprene synthase.

### 3.4 Correlation analysis of *TPS* gene expression changes among susceptible and resistant *M. quinquenervia* to *A. psidii*

**FIGURE 4:**
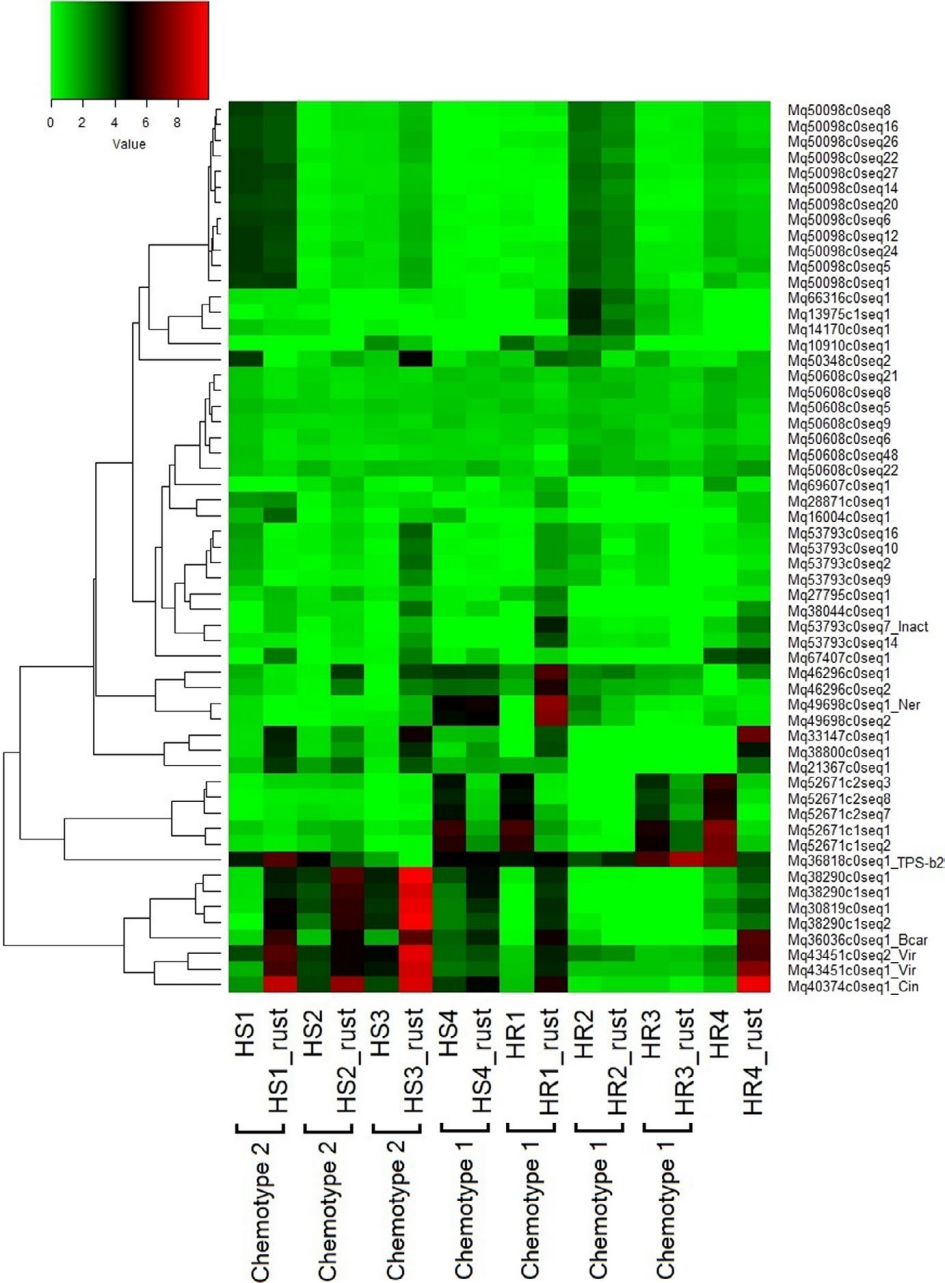
Heatmap of abundance of gene expressions (transcript per million; TPM) of 57 *TPS* transcripts observed in highly susceptible (HS) and highly resistant (HR) *Melaleuca quinquenervia* before and 5 days post *Austropuccinia psidii* inoculation (“_rust”). Results from functional characterization and terpene profiling were added after transcript IDs and sample names, respectively. Inact, inactive putative TPS; Ner, nerolidol synthase; TPS‐b2, terpene synthase of clade b2; Bcar, β‐caryophyllene synthase; Vir, viridiflorol synthase; Cin, 1,8‐cineole synthase. TPM values were log_2_‐transformed. Cells in red, black, and green represent high, moderate, and low expressions of a transcript in each sample

We hierarchically clustered the overall expression pattern of putative *TPS* transcripts and characterized *TPS* genes in highly susceptible (HS) and highly resistant (HR) *M. quinquenervia* plants (Figure 4). In addition, to assess whether samples with the same chemotype correlated with each other in changes of *TPS* gene expression after *A. psidii* infection, we evaluated the correlation of gene expression FC of *TPS* transcripts among the eight samples (Figure S3).

Overall, we observed that *M. quinquenervia* plants from each chemotype expressed more of the *TPS* genes responsible for its dominant terpenes at 5 dpi compared with the other chemotype, regardless of resistance and susceptibility status (Figure 4). For example, Chemotype 2 plants (all highly susceptible, HS1, HS2, and HS3) expressed the 1,8‐cineole synthase gene (Mq40374c0seq1_Cin) and the viridiflorol synthase gene (Mq43451c0seq2_Vir) more strongly at 5 dpi than Chemotype 1 plants, which had one highly susceptible (HS4) plant in addition to highly resistant (HR) plants (Figure 4; Table S2).

### 3.5 Terpene composition in control and *A. psidii*‐infected *M. quinquenervia* and chemotype determination

**FIGURE 5:**
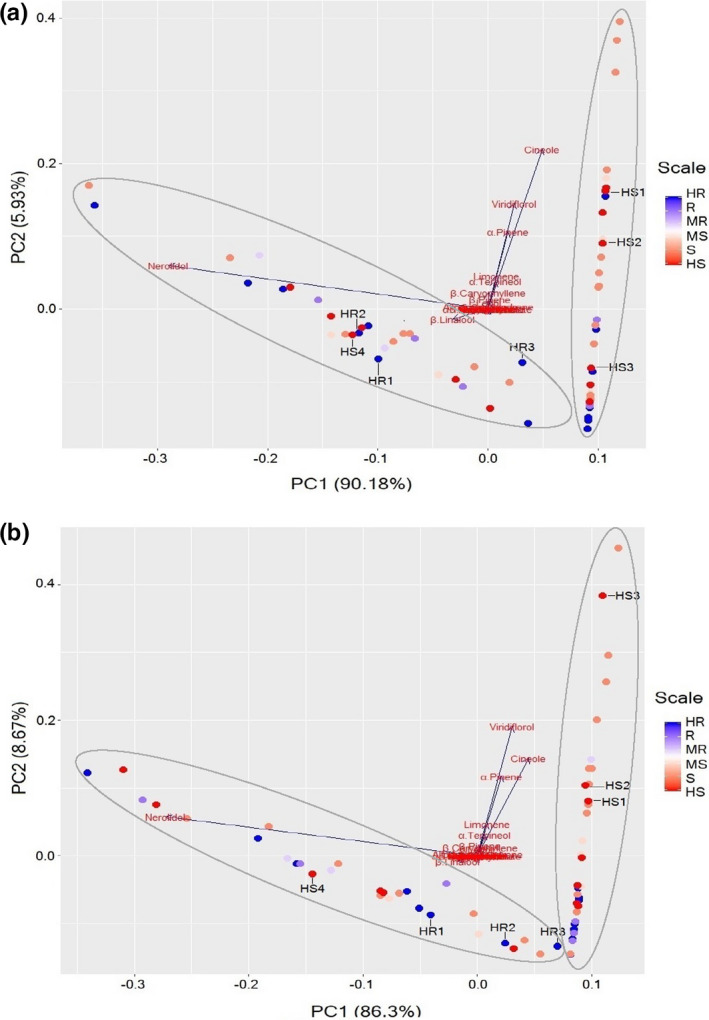
Principal component analysis bi‐plot of terpene compositions of 62 *Melaleuca quinquenervia* plants (a) before *Austropuccinia psidii* infection and (b) 5 days’ postinoculation with *A. psidii* (5 dpi). The loadings vectors show dominant features (in this case terpene concentration) that contribute to variation between samples. The color gradient scale indicates the disease score of each sample (dot). Dots labeled with HS1, HS2, HS3, HS4, HR1, HR2, and HR3 are samples analyzed by RNASeq. Gray ellipsoids indicate chemotypes, with Chemotype 1 along the *x*‐axis and Chemotype 2 along the *y*‐axis

Variation in terpene composition among 62 *A. psidii*‐screened *M. quinquenervia* plants was determined by PCA. This was to test whether chemotypic variation contributed to the resistance of the plants to *A. psidii* infection. The PCA plots of terpene profiles of plants from before and after *A. psidii* infection (Figure 5) showed that two chemotypes were present in these *M. quinquenervia* samples. The first two principal components of the analysis explained most of the variance from the samples (96.1% before infection and 95.0% after infection, respectively). Following Ireland et al. (2002), we assigned the cluster dominated by nerolidol as Chemotype 1, and the other cluster with dominant features of α‐pinene, limonene, 1,8‐cineole, α‐terpineol, β‐caryophyllene, and viridiflorol as Chemotype 2 (Figure 5). Chemotypes contained similar numbers of individuals with 29 Chemotype 1 and 33 Chemotype 2 individuals.

Chemotype did not explain resistance against *A. psidii—*susceptible and resistant plants of varying degrees were distributed across both chemotypes. A trend of resistant plants (samples scored as HR, R, and MR) from Chemotype 2 aggregating toward the lower end (negative PC2 values) of the cluster was visible, both before and after *A. psidii* infection compared to susceptible plants (HS, S, and MS; Figure 5).

### 3.6 Differences in terpene concentration between resistant and susceptible *M. quinquenervia* plants of Chemotypes 1 and 2 after *A. psidii* infection

We calculated the proportional increase and decrease of terpene concentrations for resistant and susceptible plants in both chemotypes. In *M. quinquenervia* Chemotype 1, the most abundant terpene observed was nerolidol (Table S3). Resistant (RES) plants had a higher concentration of nerolidol on average compared with susceptible (SUS) plants both before and after *A. psidii* infection with decreasing concentrations 5 dpi (Table S3; Figure S4). Rust infection, susceptibility, or interaction of both did not significantly alter terpenes in Chemotype 1 plants (Table 1).

**TABLE 1:** Two‐way ANOVA (Type III sums of squares) that tested significance in changes of terpenes between resistant and susceptible *Melaleuca quinquenervia* Chemotype 1 plants, before and after *Austropuccinia psidii* infection

Chemotype 1	Residual	Phenotype			Infection			Phenotype × infection		
	*df*	*df*	*F*	*p*‐value	*df*	*F*	*p*‐value	*df*	*F*	*p*‐value
α‐Pinene	54	1	0.37	.54	1	0.08	.78	1	0.27	.60
Limonene	54	1	0.14	.71	1	1.33	.25	1	0.64	.43
1,8‐Cineole	54	1	0.06	.80	1	0.00	.95	1	0.00	.95
β‐Linalool	54	1	1.74	.19	1	2.52	.12	1	0.19	.67
α‐Terpineol	54	1	0.05	.82	1	2.04	.16	1	0.05	.82
β‐Caryophyllene	54	1	2.00	.16	1	0.16	.69	1	0.41	.52
Nerolidol	54	1	0.27	.60	1	1.06	.31	1	0.01	.91
Viridiflorol	54	1	1.24	.27	1	1.24	.27	1	1.24	.27
Ledol	54	1	0.02	.89	1	0.93	.34	1	0.33	.57
Σ Monoterpenes	54	1	2.16	.15	1	2.45	.12	1	0.20	.66
Σ Sesquiterpenes	54	1	0.33	.57	1	1.09	.30	1	0.01	.93
Σ Terpenes	54	1	0.78	.38	1	1.82	.18	1	0.00	.98

Note

“Phenotype” represents resistance or susceptibility.

**FIGURE 6:**
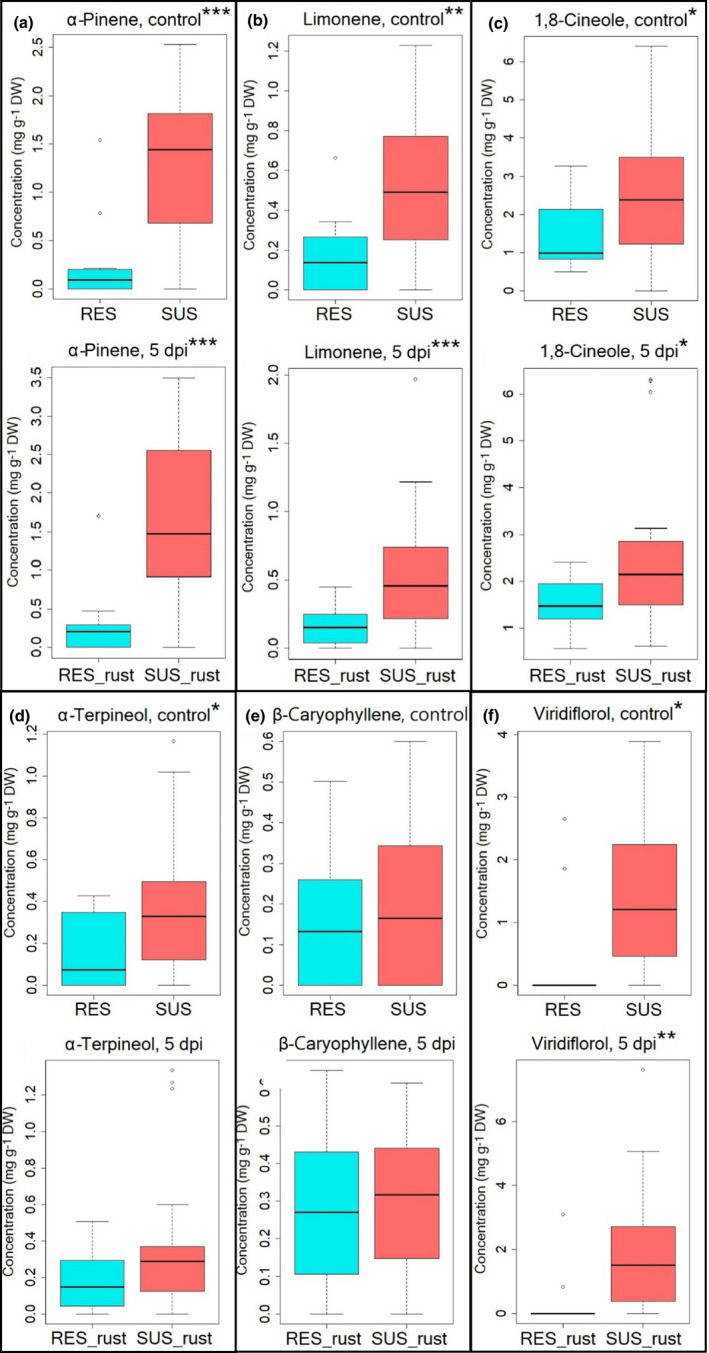
Box plots of foliar terpene concentration in Chemotype 2 *Melaleuca quinquenervia* before and 5 days’ postinoculation with *Austropuccinia psidii*. This figure shows concentrations of (a) α‐pinene, (b) limonene, (c) 1,8‐cineole, (d) α‐terpineol, (e) β‐caryophyllene, and (f) viridiflorol. RES, samples scored as resistant; SUS, samples scored as susceptible. “_rust” represents samples after *A. psidii* infection. *p < .05, **p < .01, ***p < .001 using Welch's *t* test

In contrast, Chemotype 2 susceptible and resistant plants showed significant differences in most of the dominant terpene compounds, wherein susceptible (SUS) plants had significantly higher concentrations of α‐pinene, limonene, 1,8‐cineole, and viridiflorol than resistant (RES) plants under both controlled conditions and at 5 dpi (Table S4; Figure 6). Overall, susceptible (SUS) plants had more monoterpenes and sesquiterpenes both before and at 5 dpi, as well as a higher proportional increase, than resistant (RES) plants (Table 2). Both SUS and RES plants of Chemotype 2 in general contained relatively more 1,8‐cineole than viridiflorol (Table 2). Rust infection significantly increased the concentration of β‐caryophyllene in *M. quinquenervia* plants (Table S4). The concentration of α‐terpineol was only significantly higher in susceptible plants compared with resistant plants prior to inoculation (Figure 6d), and the concentration of β‐caryophyllene was not significantly different between resistant and susceptible plants at control or 5 dpi (Figure 6e). A striking difference was the lack of viridiflorol observed in most resistant (RES) plants in Chemotype 2 (Figure 6f). Two outliers produced viridiflorol, but in the remaining nine resistant (RES) plants, no viridiflorol was detected.

**TABLE 2:** Two‐way ANOVA (Type III sums of squares) that tested significance in changes of terpenes between resistant and susceptible *Melaleuca quinquenervia* Chemotype 2 plants, before and after infection by *Austropuccinia psidii*

Chemotype 2	Residual	Phenotype			Infection			Phenotype × infection		
	*df*	*df*	*F*	*p*‐value	*df*	*F*	*p*‐value	*df*	*F*	*p*‐value
α‐Pinene	62	1	28.95	<.0001***	1	0.54	.46	1	0.31	.58
Unknown	62	1	4.05	.05*	1	0.08	.78	1	0.08	.78
β‐Pinene	62	1	8.58	.005**	1	0.11	.75	1	0.41	.53
Limonene	62	1	16.78	.0001***	1	0.15	.70	1	0.30	.59
1,8‐Cineole	62	1	7.39	.01**	1	0.01	.93	1	0.03	.87
ϒ‐Terpinene	62	1	0.02	.88	1	3.60	.06	1	0.06	.81
Terpinen‐4‐ol	62	1	2.07	.16	1	2.07	.16	1	2.07	.16
α‐Terpineol	62	1	5.62	.02*	1	0.06	.80	1	0.00	.96
α‐Terpineol acetate	62	1	0.49	.49	1	0.49	.49	1	0.49	.49
α‐Gurjunene	62	1	2.07	.16	1	2.07	.16	1	2.07	.16
β‐Caryophyllene	62	1	0.11	.74	1	4.35	.04*	1	0.01	.94
α‐Caryophyllene	62	1	0.20	.65	1	0.20	.65	1	2.21	.14
Naphthalene	62	1	0.49	.49	1	0.49	.49	1	0.49	.49
Alloaromadendrene	62	1	1.84	.18	1	0.15	.70	1	0.49	.48
Varidiflorene	62	1	0.04	.85	1	0.97	.33	1	0.08	.78
δ‐Cadinene	62	1	0.59	.45	1	1.05	.31	1	0.19	.67
Viridiflorol	62	1	11.22	.001**	1	0.40	.53	1	0.61	.44
Ledol	62	1	2.79	.10	1	0.67	.42	1	0.33	.57
Σ Monoterpenes	54	1	17.88	<.0001***	1	0.10	.75	1	0.03	.86
Σ Sesquiterpenes	54	1	10.47	.002**	1	1.02	.32	1	0.47	.50
Σ Terpenes	54	1	17.41	<.0001***	1	0.40	.53	1	0.17	.69

Note

“Phenotype” represents resistance or susceptibility.

* *p* < .05

** *p* < .01

*** *p* < .001.

### 3.7 Integrated investigation of terpenes and *TPS* gene expressions from highly susceptible and highly resistant *M. quinquenervia* after *A. psidii* infection

Combining the results from terpene profiling, RNASeq differential gene expression, and functional characterization of *TPS* genes from the seven highly susceptible (HS) and highly resistant (HR) *M. quinquenervia* samples, we observed that inductions in most of the characterized *TPS* genes after *A. psidii* infection were not positively associated with foliar terpene concentrations (Tables S2, S5 and S6).

For example, HR1 had the nerolidol synthase gene (Mq49698c0seq1_Ner) most strongly expressed and induced 5 dpi (207‐fold) compared with all other plants (Table S2), but showed a decrease in nerolidol, and the highest concentration of the terpene at 5 dpi compared with other plants of Chemotype 1 (Table S5), indicating that other nerolidol synthases may be responsible for the high constitutive amounts found prior to rust inoculation.

Changes in terpene concentrations after *A. psidii* infection among HS individuals were variable; in one individual (HS3), all terpenes increased at 5 dpi, and the total terpene concentration of HS3 at 5 dpi was higher than the other two individuals (Table S6). The three characterized terpene synthase genes—1,8‐cineole synthase gene (Mq40374c0seq1_Cin), viridiflorol synthase gene (Mq43451c0seq2_Vir), and β‐caryophyllene synthase gene (Mq36036c0seq1_BCar)—were most abundantly expressed at 5 dpi in HS3 compared with HS2 and HS3 (Table S2).

## 4 DISCUSSION

The role of terpenes in defense of woody trees against fungal infection is complex partly because of the background variation in constitutive foliar terpenes within a single species. This variation is extreme within Myrtaceae with chemotypic variation occurring widely (Keszei et al., 2008; Padovan et al., 2014). Recent attempts to correlate terpene profiles with resistance to rust in *Eucalyptus* concluded that a deeper study of terpenes was necessary (Yong et al., 2019).

### 4.1 Functional identification of terpene synthases through transcriptome and metabolite profiling

The combination of metabolite and transcriptome profiling in this study allowed us to identify induced expression of *TPS* genes in response to *A. psidii*. Six new *TPS* genes were functionally characterized (Figure 2), which increased the number of existing *TPS* genes that had been characterized in Myrtaceae by 30%. Furthermore, some of these *TPS* genes showed expression profiles that correlated with metabolite data, which are rare in comparative studies of transcriptome and metabolite profiling of terpenes. We also observed that although no particular chemotype contributed resistance to *A. psidii*, Chemotype 2 susceptible plants contained significantly more constitutive terpenes characteristic of Chemotype 2 than Chemotype 2 resistant plants. These results contrast with those of Yong et al. (2019), who found that combinations of several terpenes correlated to resistance in *E. globulus* and *E. obliqua* although the significant terpenes differed between the species.

Six new *TPS* genes have been functionally characterized in this study (Figure 2). Terpene synthase genes from species of Myrtaceae have been shown to be difficult to functionally characterize (Keszei et al., 2010; Külheim et al., 2015). Despite Myrtaceae being a large family of around 5650 species with each species estimated to have between 25 and 120 *TPS* genes, only 15 of them had been functionally characterized before this study (Keszei et al., 2010; Külheim et al., 2015; Padovan et al., 2010, 2017; Sharkey et al., 2013).

### 4.2 Myrtle rust induces *TPS* and alters terpene concentrations in *M. quinquenervia*

To our knowledge, this is the first study in Myrtaceae that showed induced expression of *TPS* genes with biotrophic fungal infection (Figure 4; Table S2). Induction of *TPS* genes or changes of terpene concentrations in response to biotic and abiotic stress has rarely been shown in Myrtaceae (Webb et al., 2014). Apart from a recent study where some *TPS*‐encoding genes and terpenes were observed to be upregulated in *E. grandis* upon challenge by *Leptocybe invasa* (gall wasp; Oates et al., 2015) and another study which found induced expression of isoprene synthase‐ and β‐caryophyllene synthase‐encoding genes (*EgrTPS084* and *EgrTPS038*, respectively) in *E. grandis* in response to *C. austroafricana* infection (Visser et al., 2015), there has been little to no evidence of induction of terpenes in *Eucalyptus* or *Melaleuca* in response to wounding, herbivory, or even application of methyl jasmonate (MeJA; Henery et al., 2008). Henery et al. (2008) noted that it was possible that herbivory and MeJA could still induce *TPS* genes in *Eucalyptus* without increasing the amount of constitutive terpenes contained in the leaves due to volatile losses. This is the pattern found through studies of monoterpene synthase activity assay and terpene profiling in *Pinus ponderosa*, *P. contorta*, *and Abies concolor* (Litvak & Monson, 1998). However, Bustos‐Segura and Foley (2018) found that headspace volatiles in *M. alternifolia* were minor in undamaged plants. Henery et al. (2008) argued that evergreen trees such as Myrtaceae are more likely to have constitutively expressed terpenes for defense against insect herbivores, whereas deciduous trees tend to use induction of terpenes for such defense. However, in contrast to Henery et al. (2008), we were able to identify multiple *TPS* genes that were induced in *M. quinquenervia*, in response to fungal attack, suggesting that these plants may have utilized induction of terpenes as one of the modes of defense.

Several *TPS* genes had changes in gene expression that correlated with changes in concentration of respective terpenes (Table S6). Of the eight plants analyzed by RNASeq, this was observed in one individual of Chemotype 2 (susceptible plant individual HS3). In HS3 after rust infection, the induction of the 1,8‐cineole synthase gene (Mq40374c0seq_Cin), viridiflorol synthase genes (Mq43451c0seq1_Vir, Mq43451c0seq2_Vir), and a β‐caryophyllene synthase gene (Mq36036c0seq1_BCar) mirrored the increase of the individual's foliar concentrations of 1,8‐cineole, viridiflorol, and β‐caryophyllene (Tables S2 and S6). Although a positive trend was observed in this individual, other individuals showed discrepancies between induction of *TPS* genes and concentrations of terpenes, for example, nerolidol synthase and nerolidol (Tables S2 and S5). One possible explanation for this discrepancy may be transcriptional variation. Nie et al. (2006) showed that correlation between transcript and corresponding protein explained <30% of variation in corresponding protein abundance through the *Desulfovibrio vulgaris* model and that certain functional gene categories showed greater variation. A second explanation could be that the initial enzyme product had undergone further modifications such as hydroxylation or glycosylation (Aharoni et al., 2004). For example, certain types of terpenes may be more likely to be glycosylated than other types—the study of *Vitis vinifera* berries by Wen et al. (2015) observed that free‐form nerol was less abundant than its glycosylated‐bound form (neryl glycoside) compared with linalool, where its free form was more abundant than its glycosylated form (linaloyl glycoside). Furthermore, glycosylation has been suggested to function to stabilize terpenes as inactive, nontoxic forms for plants to store at high concentrations. Plants could then react to the pathogen through reactivation of the terpene by glycosidases and de‐compartmentation to bring the toxic terpene form in contact with the pathogen (Vogt & Jones, 2000; Wittstock & Gershenzon, 2002). A recent study found that two families of UDP‐glycosyltransferase (UGT) genes were significantly associated with terpene concentration in a genome‐wide association study in *E. polybractea* (Kainer et al., 2019). All *Eucalyptus* secretory cavities contain glycosylated terpenes although these are predominately monoterpene derivatives. The two gene families identified in *E. polybractea* (UGT76G1 and UGT85A2) have been shown to glycosylate a wide range of terpenes in vitro (Caputi et al., 2008). Although we would expect levels of homology between *Eucalyptus* and *Melaleuca*, we did not find UGT76G1 and UGT85A2 homologues in the *M. quinquenervia* transcriptome.

### 4.3 Chemotype did not affect resistance to myrtle rust, but higher concentration of terpenes could induce spore germination

Chemotype differences alone did not seem to affect the growth of *A. psidii* on *M. quinquenervia* (Figure 5). This finding was consistent with a study from Florida where *M. quinquenervia* is an invasive weed (Rayamajhi et al., 2010). Both Chemotype 1 (dominant in nerolidol) and Chemotype 2 (dominant in viridiflorol) *M. quinquenervia* in Florida showed spore development of *A. psidii* which was classified in the genetic cluster of C4. The C4 biotype is considered as the same “Pandemic biotype” as the biotype identified in Australia through microsatellite marker genotyping (da S. Machado et al., 2015; Rayamajhi et al., 2010; Sandhu et al., 2016; Stewart et al., 2018).

We have previously shown that in *M. alternifolia*, chemotype had a small effect on myrtle rust susceptibility with plants containing higher levels of 1,8‐cineole being more susceptible (Bustos‐Segura et al., 2015). This is similar to susceptible *M. quinquenervia* in that Chemotype 2 produced significantly higher concentrations of total terpenes and constitutive terpenes such as viridiflorol and those in the “cineole cassette” (α‐pinene, limonene, and 1,8‐cineole) at control and 5 dpi compared with resistant *M. quinquenervia* (Table 2; Figure S2; Fahnrich et al., 2011). Rayamajhi et al. (2010) also detected higher concentrations of total terpenes and limonene, myrcene, and β‐caryophyllene in susceptible *M. quinquenervia* compared with resistant *M. quinquenervia*, although it was unclear which chemotype was studied. We can speculate that higher concentrations of these constitutive terpenes in Chemotype 2 susceptible plants may have acted as a stimulant to rust spore germination. The diffusion assay study by French (1961) found that limonene strongly stimulated the germination of *Puccinia graminis* var. *tritici* (stem rust) uredospores, with an activity rating of 128 at 10^−3^ dilution, which was higher than twelve other types of terpenes. α‐Pinene was also rated as stimulatory (rating of 73 at 10^−3^ dilution; French, 1961). Similarly, the study by Rodríguez et al. (2011) demonstrated that downregulated expression of a (+)‐limonene synthase gene (*CitMTSE1*) in transgenic *Citrus sinensis* (orange) resulted in decreased limonene, which in turn made *C. sinensis* less likely to be infected by *Penicillium digitatum* (green mold), thereby suggesting that limonene accumulation may be involved in successful fungal development. In addition, Droby et al. (2008) also examined stimulatory effects of citrus volatiles and found that limonene strongly enhanced the germ tube elongation of *P. digitatum*, followed by α‐pinene which moderately stimulated *P. digitatum* growth. One possible reason for the stimulatory effects of these terpenes on *A. psidii*, as French (1961) postulated, may be that rust uredospores required a stimulatory substrate to activate certain components necessary for germination, since the rust themselves seemed to have lipophilic self‐inhibitors. Therefore, in our study, limonene and α‐pinene may have promoted the germination of *A. psidii* uredospores. However, the molecular mechanisms of stimulatory effects of terpenes on rust spores would require further study.

## 5 CONCLUSION

This study showed that mechanisms of resistance to *A. psidii* in *M. quinquenervia* may require a concerted effort of multiple defense products in addition to terpenes and that such mechanisms of resistance may vary on an intraspecific level reflecting more than terpene chemotype differences. Although terpenes are generally regarded as important components in plant resistance against pests and pathogens (Gershenzon & Dudareva, 2007), constitutive terpenes and regulation of *TPS* genes may not be causative factors for the resistance of *M. quinquenervia* to *A. psidii* infection; mixes of other volatile compounds may be required to act synergistically to confer resistance (Henery et al., 2008; Potts et al., 2016). Moreover, plants, including *Eucalyptus*, often recruit a broad range of defense products via complex regulatory networks in response to infection (Naidoo et al., 2014) or induced defenses such as reinforcement of the cell walls as shown through induction of genes related to cell wall biosynthesis in *Syzygium luehmannii* (Tobias et al., 2018) and cuticular wax composition and structure in various *Eucalyptus* spp. (Santos et al., 2019). *M. quinquenervia* may utilize a combination of these constitutive or inducible defense‐related factors in addition to volatile compounds to achieve effective resistance against the rust fungus *A. psidii*, and such a combination may not be identical among individuals of *M. quinquenervia*.

## CONFLICT OF INTEREST

The authors declare that there is no conflict of interest.

## AUTHOR CONTRIBUTION

J‐FH, WJF, and CK conceived the study. J‐FH performed the gene expression study and selected candidate genes for functional characterization with the assistance of CK. J‐FH performed RNA extractions. SK and JD performed the cloning and functional characterization of the genes. J‐FH performed the GC‐MS experiment and analyzed the data with DK. J‐FH wrote the first draft of the manuscript, CK and WJF edited the manuscript, and all authors read and approved the manuscript prior to submission.

## Supporting information

Supplementary Material

## Data Availability

RNAseq data were deposited to the NCBI short‐read archive (SRA) with the identifier SRP095052 and BioProject accession number PRJNA357284. All remaining data for this study can be found in the supplemental material.
