# Supplementary material for: Characterization of terpene biosynthesis in Melaleuca quinquenervia and ecological consequences of terpene accumulation during myrtle rust infection
Source: Plant Environ Interact. 2021 Jun 24;2(4):177–93. doi: 10.1002/pei3.10056 (PMC10168048; doi:10.1002/pei3.10056)
Supplement: Supplementary file 1 — Supplementary Material [file PEI3-2-177-s001.pdf]

SUPPLEMENTARY MATERIALS

Figure S1. Amino acid sequences of functionally characterised *TPS* genes in *M. quinquenervia*. Sequences MqTPS1_Vir and MqTPS2_Vir are characterised viridiflorol synthases by Padovan et al. (2010) which were used as references. Sequences were aligned in ClustalW (BLOSUM62) in BioEdit and were manually curated. Amino acids shaded in dark blue and light green represents identical/highly similar and similar amino acids aligned, respectively. Underlined regions show conserved motifs. Ner: Nerolidol synthase; BCar: β-Caryophyllene synthase; Cin: 1,8-Cineole synthase; w/oSP: without signal peptide; TPS-b2: TPS-b2 synthase (likely to be an isoprene synthase); Inact: Inactive terpene synthase.

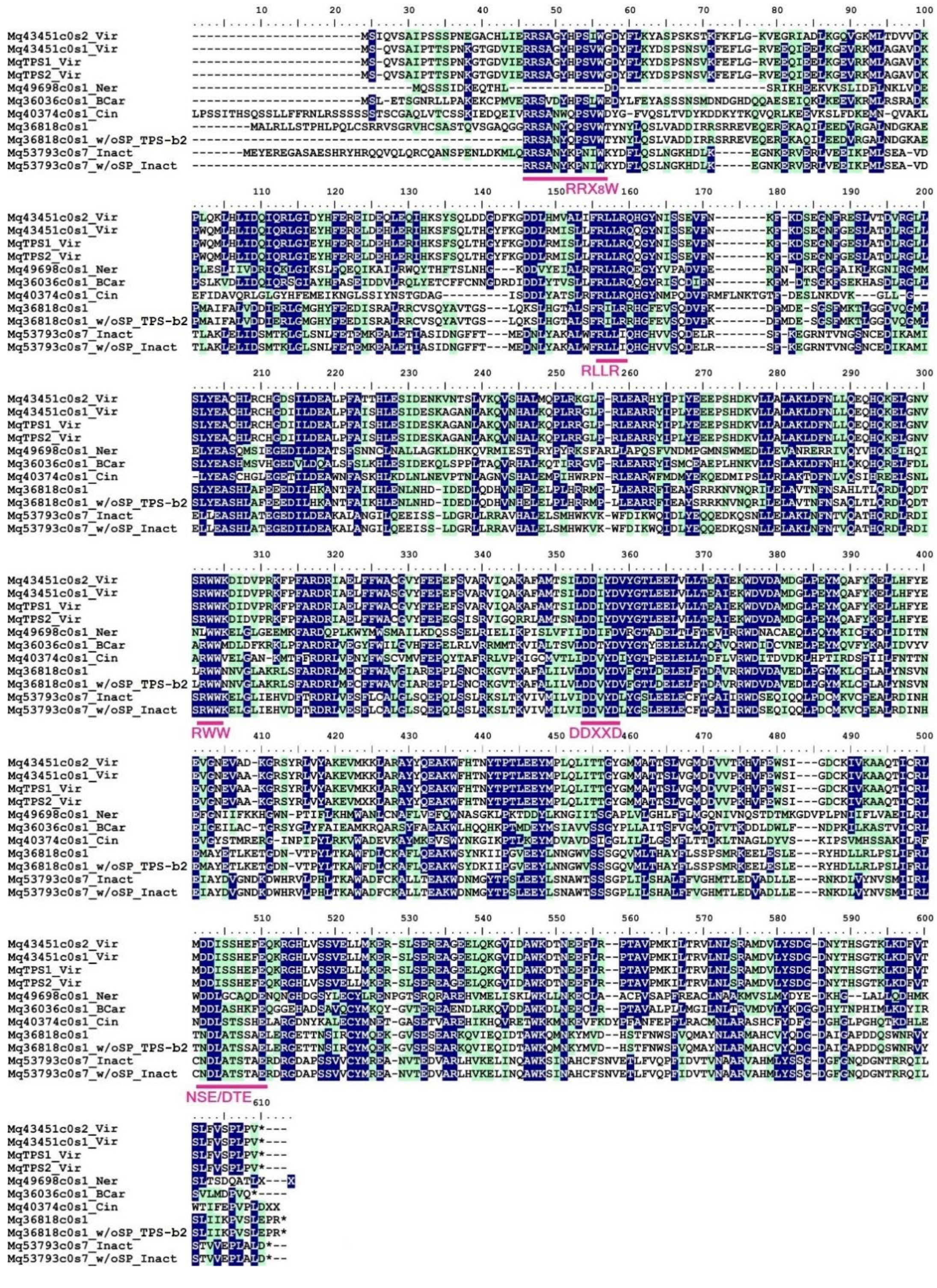
Mq43451c0s2_Vir
Mq43451c0s1_Vir
MqTPS1_Vir
MqTPS2_Vir
Mq49698c0s1_Ner
Mq36036c0s1_BCar
Mq40374c0s1_Cin
Mq36818c0s1
Mq36818c0s1_w/oSP_TPS-b2
Mq53793c0s7_Inact
Mq53793c0s7_w/oSP_Inact
RRX8W
RLLR
RWW
DDXXD
NSE/DTE

Fig. S2. Amino acid sequences of the functionally characterised *TPS-b2* gene in *M. quinquenervia*, with characterised isoprene synthase of *M. alternifolia* (Genbank #AY279379) (Sharkey et al. 2013) and predicted isoprene synthase of *E. grandis* (Külheim et al. unpublished). Sequences were aligned in ClustalW (BLOSUM62) in BioEdit and were manually curated. Amino acids shaded in dark blue and light green represents identical/highly similar and similar amino acids aligned, respectively. Boxed regions in magenta show the four amino acids that form a combination (FSFN) canonical to isoprene synthases.

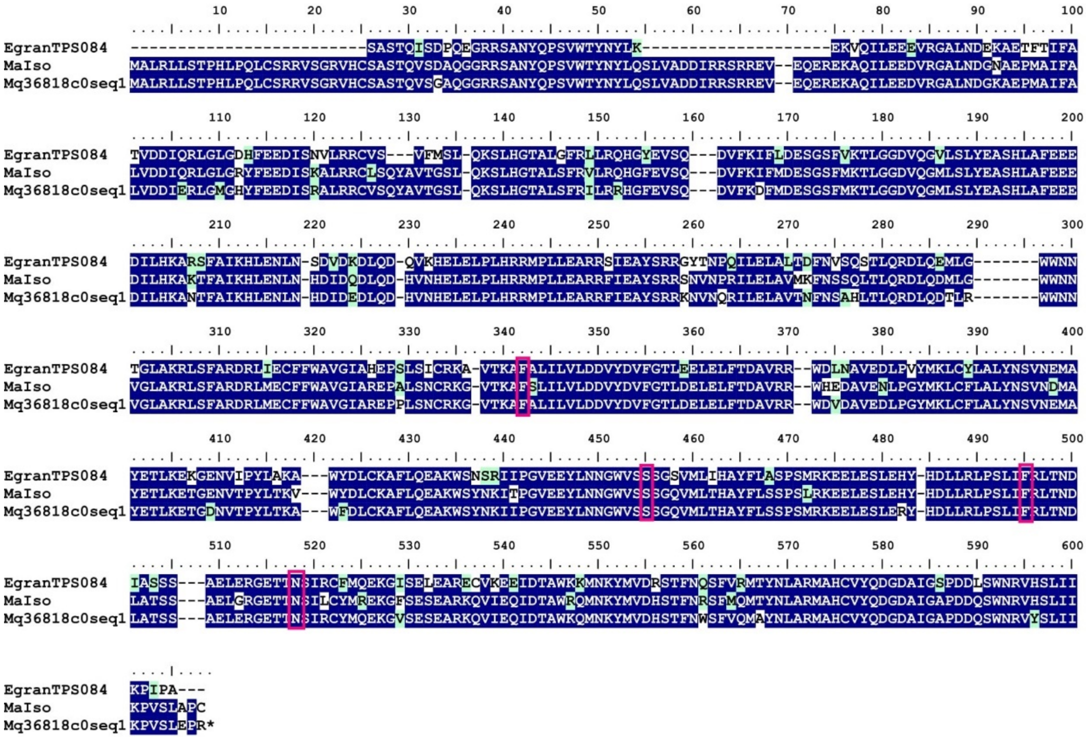

Figure S3. Pearson's correlation plot of gene expression fold-changes of fifty-seven *TPS* transcripts between highly susceptible (HS) and highly resistant (HR) *M. quinquenervia* plants. Fold-change values were $\log_2$-transformed. *$P < 0.05$, **$P < 0.01$, *** $P < 0.001$.

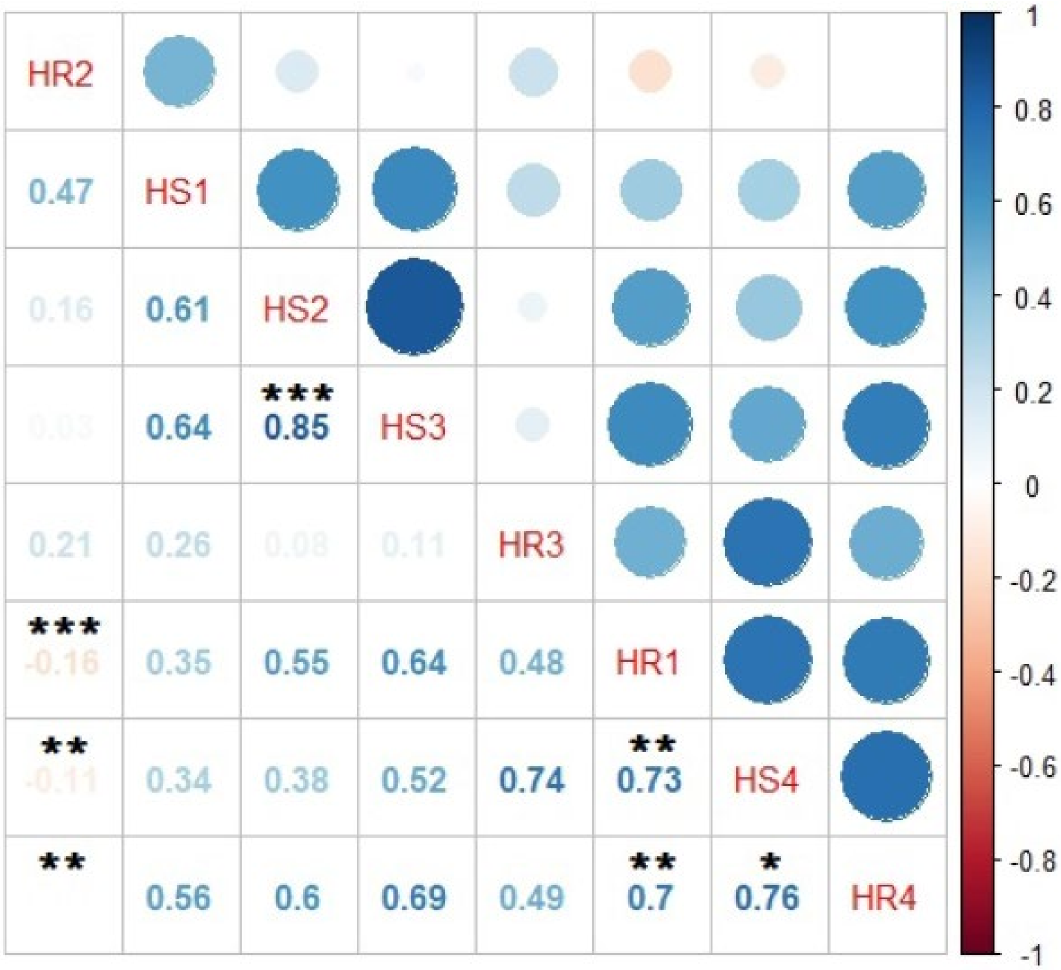

Figure S4 Welch's *t*-test examining the differences between resistant and susceptible *M. quinquenervia* plants in concentrations of major terpene compounds in chemotype 1, which include (A, B) β-linalool and (C, D) nerolidol. RES: Samples scored as highly resistant (HR), resistant (R) and moderately resistant (MR); SUS: Samples scored as highly susceptible (HS), susceptible (S) and moderately susceptible (MS). '_rust' represents samples after *A. psidii* infection.

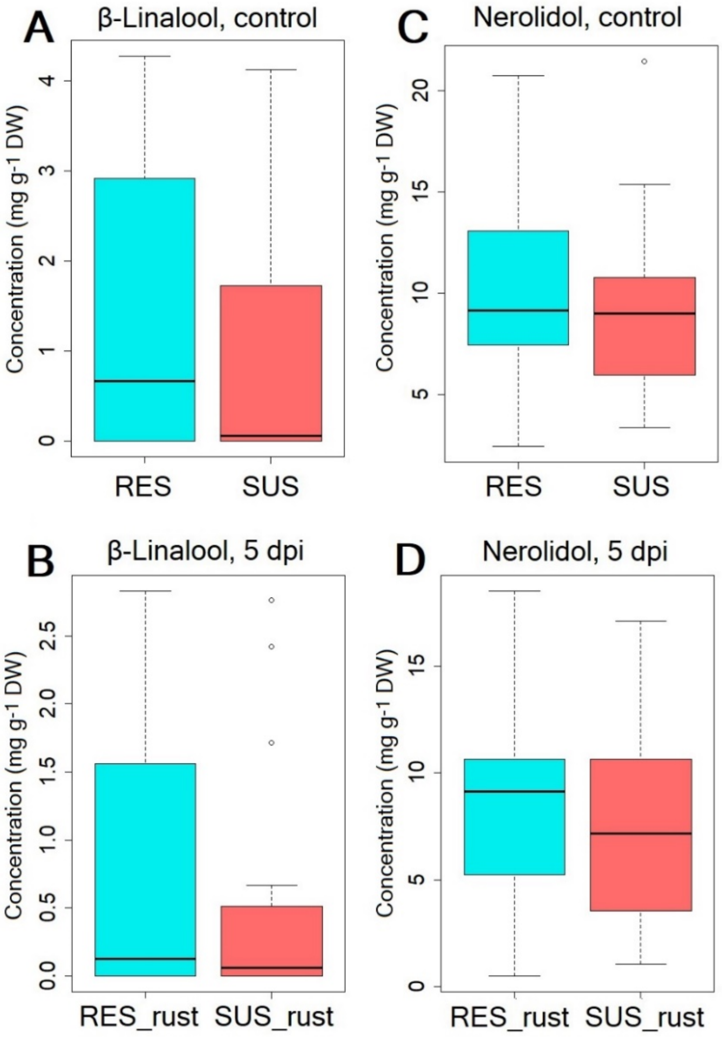

Table S1. Primers used in this study

**Primer name**	**Sequence**	**Orientation**	**Use**
MqTPS136036 fwd	ATGTCTCTAGAAACCTCAGG	fwd	Isolation
MqTPS136036 Ibaf	ATGGTAGGTCTCAGCGCATGTCTCTAGAAACCTCAGGAAATC	fwd	Cloning into pASK-Iba37+, digest with BsaI
MqTPS136036 Ibar	ATGGTAGGTCTCATATCATTGCACTGGGTCCATGAGCACC	rev	Cloning into pASK-Iba37+, digest with BsaI
MqTPS136036 rev	CTATTGCACTGGGTCCATG	rev	Isolation
MqTPSa434511 fwd	ATGTCGCAAGTTTCAGCGATTC	fwd	Isolation
MqTPSa434511 Ibaf	ATGGTAGGTCTCAGCGCATGTCGCAAGTTTCAGCGATTCC	fwd	Cloning into pASK-Iba37+, digest with BsaI
MqTPSa434511 Ibar	ATGGTAGGTCTCATATCACACCGGCAAGGGGCTAACGAA	rev	Cloning into pASK-Iba37+, digest with BsaI
MqTPSa434512 fwd	ATGTCAATTCAAGTTTCG	fwd	Isolation
MqTPSa434512 Ibaf	ATGGTAGGTCTCAGCGCATGTCAATTCAAGTTTCGGCAATTC	fwd	Cloning into pASK-Iba37+, digest with BsaI
MqTPSa434512 Ibar	ATGGTAGGTCTCATATCACACCGGCAAGGGGCTAACGAA	rev	Cloning into pASK-Iba37+, digest with BsaI
MqTPSa434512 rev	TCACACCGGCAAGGGGCTAAC	rev	Isolation
MqTPSb236818 Ibaf	GGTACCATGGCACTTCGCCTTTTGTC	fwd	Cloning into pASK-Iba37+, digest with PstI+KpnI
MqTPSb236818 Ibaf-2	GGTACCCGCAGATCGGCCAATTATC	fwd	Expression (without signal peptide)
MqTPSb236818 Ibar	CTGCAGTCAACGTGGCTCAAGAGA	rev	Cloning into pASK-Iba37+, digest with PstI+KpnI
MqTPSb236818 rev	TCAACGTGGCTCAAGAGAAAC	rev	Isolation
MqTPSb40374 fwd	CTCCCCTCCTCAATCACCCATAG	fwd	Isolation
MqTPSb40374 Ibaf	ATGGTAACCTGCATTAGCGCCTCCCCTCCTCAATCACCCATA	fwd	Cloning into pASK-Iba37+, digest with BspMI
MqTPSb40374 Ibaf-2	ATGGTAACCTGCATTAGCGCAGACGTTCAGCAAATTGGCAGCC	fwd	Expression (without signal peptide)
MqTPSb40374 Ibar	ATGGTAACCTGCATTATATCAATCGAGGGGCACAGGTTCAAATA	rev	Cloning into pASK-Iba37+, digest with BspMI
MqTPSb40374 rev	CTAATCGAGGGGCACAGGTTC	rev	Isolation
MqTPSb53793 fwd	ATGGAGTACGAAAGGGAAG	fwd	Isolation
MqTPSb53793 Ibaf	ATGGTAGGTCTCAGCGCATGGAGTACGAAAGGGAAGGAGC	fwd	Cloning into pASK-Iba37+, digest with BsaI
MqTPSb53793 Ibaf2	ATGGTAGGTCTCAGCGCAGAAGATCAGCAAATTATAAGCCGA	fwd	Expression (without signal peptide)
MqTPSb53793 Ibar	ATGGTAGGTCTCATATCAGTCGAGAGCAAGAGGCTCGAC	rev	Cloning into pASK-Iba37+, digest with BsaI
MqTPSb53793 rev	TCAGTCGAGAGCAAGAGGCTC	rev	Isolation
MqTPSg46296 Ibaf	ATGGTAGGTCTCAGCGCATGGCCTTGTCAAGAGCAACCTT	fwd	Cloning into pASK-Iba37+, digest with BsaI
MqTPSg46296 Ibar	ATGGTAGGTCTCATATCAGAGCAAAGACTTCATGTGGTGCT	rev	Cloning into pASK-Iba37+, digest with BsaI
MqTPSg46296 rev	TTAGAGCAAAGACTTCATGTG	rev	Isolation
MqTPSg49698 f-2	ATGCAATCGTCGAGCATTGACAAAG	fwd	Isolation - 2
MqTPSg49698 fwd	GCACATCAACCTCCCATCAAG	fwd	Isolation
MqTPSg49698 Iba2f	ATGGTAACCTGCATTAGCGCATGCAATCGTCGAGCATTGACAAA	fwd	Cloning into pASK-Iba37+, digest with BspMI
MqTPSg49698 Iba2r	ATGGTAACCTGCATTATATCAAAGTGTCGCCTGATCAGAGGTC	rev	Cloning into pASK-Iba37+, digest with BspMI
MqTPSg49698 Iba9f	ATGGTAACCTGCATTAGCGCATCGCCCGGTTCTTCACGAGC	fwd	Cloning into pASK-Iba37+, digest with BspMI
MqTPSg49698 Iba9r	ATGGTAACCTGCATTATATCAAAGTGTCGCCTGATCAGAGGTC	rev	Cloning into pASK-Iba37+, digest with BspMI
MqTPSg49698 r-2	CTAAAGTGTCGCCTGATCAGAGGTC	rev	Isolation - 2
MqTPSg49698 rev	CTAAAGTGTCGCCTGATCAG	rev	Isolation

Table S2. Expression abundance (TPM) and fold-change (FC) of eight candidate *TPS* genes selected for functional characterisation in highly susceptible (HS) and highly resistant (HR) *M. quinquenervia* samples (four biologically independent samples each) after *A. psidii* infection. Results from functional characterisations are added after transcript IDs. BCar: β-Caryophyllene synthase; TPS-b2: Terpene synthase of clade b2 (likely to be an isoprene synthase); Cin: 1,8-Cineole synthase; Vir: Viridiflorol synthase; Ner: Nerolidol synthase; inact: inactive putative terpene synthase. Symbols following transcript IDs signify significant differential expressions (DE) of corresponding transcripts detected by edgeR and DESeq2 (methods detailed in Hsieh et al. 2017). The black dot (˙) denotes DE of the transcript in HS vs. HR, and asterisks (*) denote DE of the transcript in HS vs. HS_rust. 'TPM (control)' and 'TPM (5 dpi)' are TPM values added below corresponding FC values as accompanying information. An arbitrary value of 1 was added before calculating FC.

Transcript ID	HS1 FC	HS2 FC	HS3 FC	HS4 FC	HR1 FC	HR2 FC	HR3 FC	HR4 FC
**Mq36036c0seq1_BCar***	**36.81**	**15.16**	**30.31**	**2.32**	**35.82**	**0.57**	**0.97**	**81.03**
TPM (control)	0.79	1.43	2.38	6.95	0.11	0.77	0.04	0.18
TPM (5 dpi)	64.90	35.89	101.45	17.45	38.80	0.00	0.00	94.55
**Mq36818c0seq1_TPS-b2**	**4.33**	**0.27**	**0.35**	**0.88**	**1.27**	**1.71**	**3.52**	**0.08**
TPM (control)	20.83	33.36	2.25	32.05	23.05	9.61	83.98	154.34
TPM (5 dpi)	93.43	8.37	0.15	28.12	29.62	17.17	297.95	11.31
**Mq40374c0seq1_Cin • ***	**88.31**	**16.74**	**49.38**	**2.06**	**21.38**	**1.18**	**0.71**	**363.99**
TPM (control)	3.51	12.61	10.47	12.28	1.31	0.40	0.62	1.12
TPM (5 dpi)	397.19	226.78	565.26	26.37	48.48	0.65	0.15	770.70
**Mq43451c0seq1_Vir***	**40.90**	**3.15**	**22.34**	**1.43**	**14.67**	**0.91**	**0.95**	**68.25**
TPM (control)	0.93	10.27	21.48	4.14	0.21	0.24	0.16	1.85
TPM (5 dpi)	77.92	34.46	501.24	6.36	16.74	0.13	0.11	193.85
**Mq43451c0seq2_Vir**	**8.80**	**2.66**	**24.33**	**1.49**	**16.62**	**0.77**	**1.11**	**23.63**
TPM (control)	9.74	12.60	25.38	5.48	0.11	3.91	0.18	2.56
TPM (5 dpi)	93.50	35.17	640.84	8.65	17.50	2.78	0.31	83.10
**Mq46296c0seq1**	**0.47**	**12.05**	**11.22**	**0.97**	**25.10**	**1.27**	**0.92**	**4.60**
TPM (control)	1.96	0.21	0.07	14.02	2.71	3.88	2.42	0.06
TPM (5 dpi)	0.38	13.53	11.01	13.56	92.11	5.19	2.13	3.89
**Mq49698c0seq1_Ner**	**0.61**	**1.07**	**2.44**	**1.21**	**207.38**	**0.40**	**0.89**	**0.77**
TPM (control)	0.64	0.08	0.14	32.37	0.00	4.68	0.30	0.64
TPM (5 dpi)	0.00	0.15	1.78	39.54	206.38	1.26	0.16	0.26
**Mq53793c0seq7_Inact**	**2.22**	**1.50**	**2.76**	**1.00**	**19.65**	**0.89**	**0.80**	**3.73**
TPM (control)	0.07	0.08	0.04	0.00	0.00	0.35	0.30	0.90
TPM (5 dpi)	1.38	0.62	1.86	0.00	18.65	0.20	0.04	6.08

Table S3. Mean concentration (mg.g^{-1} DW) and percent up- and down-regulation (% up/down) of terpenes in resistant and susceptible *M. quinquenervia* plants of chemotype 1. RES: Samples scored as highly resistant (HR), resistant (R) and moderately resistant (MR); SUS: Samples scored as highly susceptible (HS), susceptible (S), and moderately susceptible (MR). '_rust' represents samples after *A. psidii* infection; 'increased' and 'decreased' describe the overall change in terpene concentrations when percent changes cannot be calculated (values are zero).

	Chemotype 1					
	RES	RES_rust	% up/down	SUS	SUS_rust	% up/down
Number of samples	13	13		16	16	
α-Pinene	0.22	0.20	-5.50	0.16	0.20	25.55
Limonene	0.06	0.06	-11.58	0.07	0.03	-55.88
1,8-Cineole	0.13	0.13	5.89	0.11	0.11	0.00
β-Linalool	1.48	0.82	-44.38	0.91	0.54	-41.28
α-Terpineol	0.00	0.01	increased	0.00	0.01	increased
β-Caryophyllene	0.17	0.12	-30.50	0.07	0.08	18.32
Nerolidol	9.80	8.61	-12.12	9.28	7.78	-16.09
Viridiflorol	0.01	0.00	decreased	0.00	0.00	–
Ledol	0.02	0.00	decreased	0.01	0.01	-35.79
Total Monoterpenes	1.89	1.23	-35.04	1.26	0.89	-29.43
Total Sesquiterpenes	10.00	8.73	-12.67	9.35	7.87	-15.87
Total Terpenes	11.89	9.96	-16.22	10.61	8.76	-17.48

Table S4. Mean concentration (mg.g^{-1} DW) and percent up- and down-regulation (% up/down) of terpenes in resistant and susceptible *M. quinquenervia* plants of chemotype 2. RES: Samples scored as highly resistant (HR), resistant (R) and moderately resistant (MR); SUS: Samples scored as highly susceptible (HS), susceptible (S), and moderately susceptible (MR). '_rust' represents samples after *A. psidii* infection; 'increased' and 'decreased' describe the overall change in terpene concentrations when percent changes cannot be calculated (values are zero).

	Chemotype 2					
	RES	RES_rust	% up/down	SUS	SUS_rust	% up/down
Number of samples	11	11		22	22	
α-Pinene	0.27	0.31	14.02	1.29	1.56	21.17
Unknown	0.01	0.01	32.45	0.00	0.00	–
β-Pinene	0.02	0.01	-48.48	0.11	0.14	32.70
Limonene	0.18	0.17	-8.24	0.50	0.59	16.77
1,8-Cineole	1.51	1.53	1.85	2.59	2.49	-3.81
γ-Terpinene	0.00	0.02	increased	0.00	0.02	409.66
Terpinen-4-ol	0.00	0.01	increased	0.00	0.00	–
α-Terpineol	0.17	0.19	14.50	0.36	0.38	4.37
α-Terpineol acetate	0.00	0.00	–	0.00	0.01	increased
α-Gurjunene	0.00	0.01	increased	0.00	0.00	–
β-Caryophyllene	0.17	0.28	66.17	0.19	0.29	54.65
α-Caryophyllene	0.00	0.01	increased	0.00	0.00	–
Naphthalene	0.00	0.00	–	0.01	0.00	decreased
Alloaromadendrene	0.00	0.01	increased	0.02	0.02	-15.13
Varidiflorene	0.00	0.01	increased	0.01	0.01	153.44
δ-Cadinene	0.00	0.02	increased	0.02	0.02	49.13
Viridiflorol	0.41	0.36	-12.94	1.34	1.85	38.07
Ledol	0.00	0.02	increased	0.08	0.18	127.14
Total Monoterpenes	2.16	2.24	4.16	4.85	5.18	6.78
Total Sesquiterpenes	0.58	0.72	23.72	1.66	2.38	43.15
Total Terpenes	2.73	2.96	8.30	6.51	7.56	16.05

Table S5. Mean concentration (mg.g^{-1} DW) and percent up- and down-regulation (% u/d) of terpenes in resistant and susceptible *M. quinquenervia* plants of chemotype 1 that were analysed by RNA-Seq. Group: The group that the RNA-Seq samples belongs to; RES: Samples scored as resistant ; SUS: Samples scored as susceptible. '_r' represents samples after *A. psidii* infection; 'incre' (increased) and 'decre' (decreased) describe the overall change in terpene concentrations when percent changes cannot be calculated (values are zero).

	Chemotype 1											
Group	RES									SUS		
Sample	HR1	HR1_r	% u/d	HR2	HR2_r	% u/d	HR3	HR3_r	% u/d	HS4	HS4_r	% u/d
α-Pinene	0.05	0.09	79.42	0.17	0.08	-53.02	0.69	0.30	-56.89	0.00	0.00	—
Limonene	0.00	0.00	—	0.00	0.00	—	0.24	0.00	decre	0.08	0.00	decre
1,8-Cineole	0.00	0.00	—	0.00	0.00	—	0.80	0.45	-43.58	0.00	0.00	—
β-Linalool	4.28	1.02	-76.20	1.20	0.13	-89.17	0.67	0.12	-81.78	1.70	0.36	-78.75
α-Terpineol	0.00	0.00	—	0.00	0.00	—	0.00	0.10	incre	0.00	0.00	—
β-Caryophyllene	0.09	0.00	decre	0.00	0.00	—	0.54	0.00	decre	0.00	0.00	—
Nerolidol	8.48	5.23	-38.25	9.66	2.41	-75.06	2.83	0.50	-82.34	9.87	2.05	-79.19
Ledol	0.00	0.00	—	0.00	0.00	—	0.23	0.00	decre	0.00	0.00	—
Total Monoterpenes	4.33	1.11	-74.37	1.37	0.21	-84.66	2.41	0.98	-59.43	1.78	0.36	-79.74
Total Sesquiterpenes	8.56	5.23	-38.88	9.66	2.41	-75.06	3.60	0.50	-86.08	9.87	2.05	-79.19
Total Terpenes	12.89	6.34	-50.80	11.03	2.62	-76.26	6.00	1.48	-75.40	11.65	2.41	-79.28

Table S6. Mean concentration (mg.g^{-1} DW) and percent up- and down-regulation (% u/d) of terpenes in susceptible *M. quinquenervia* plants of chemotype 2 that were analysed by RNA-Seq. Group: The group that the RNA-Seq samples belongs to; SUS: Samples scored as susceptible. '_r' represents samples after *A. psidii* infection; 'incre' (increased) and 'decre' (decreased) describe the overall change in terpene concentrations when percent changes cannot be calculated (values are zero).

	Chemotype 2								
Group	SUS								
Sample	HS1	HS1_r	% u/d	HS2	HS2_r	%u/d	HS3	HS3_r	% u/d
α-Pinene	1.47	0.85	-42.31	2.07	2.75	33.21	1.68	3.50	107.50
β-Pinene	0.25	0.19	-24.88	0.13	0.00	—	0.00	0.83	incre
Limonene	0.91	0.63	-30.89	0.60	0.61	1.77	0.93	1.97	110.80
1.8-Cineole	3.06	2.57	-16.25	1.51	0.63	-58.48	0.00	0.76	incre
α-Terpineol	0.66	0.60	-8.98	0.24	0.00	decre	0.00	0.18	incre
β-Caryophyllene	0.60	0.58	-3.47	0.34	0.16	-52.75	0.00	0.27	incre
α-Caryophyllene	0.08	0.00	decre	0.00	0.00	—	0.00	0.00	—
Alloaromadendrene	0.11	0.00	decre	0.11	0.00	decre	0.00	0.19	incre
δ-Cadinene	0.15	0.00	decre	0.00	0.00	—	0.00	0.18	incre
Viridiflorol	2.43	2.05	-15.78	3.89	3.07	-21.08	1.27	7.61	497.35
Ledol	0.17	2.07	1106.34	0.17	0.00	decre	0.00	0.22	incre
Total Monoterpenes	6.36	4.83	-23.96	4.55	3.99	-12.17	2.62	7.24	176.38
Total Sesquiterpenes	3.54	4.69	32.51	4.52	3.23	-28.38	1.27	8.48	565.28
Total Terpenes	9.90	9.53	-3.76	9.06	7.23	-20.24	3.89	15.72	303.68
